# GLCCI1 Deficiency Induces Glucocorticoid Resistance via the Competitive Binding of IRF1:GRIP1 and IRF3:GRIP1 in Asthma

**DOI:** 10.3389/fmed.2021.686493

**Published:** 2021-08-24

**Authors:** Xinyue Hu, Shuanglinzi Deng, Lisha Luo, Yuanyuan Jiang, Huan Ge, Feifei Yin, Yingyu Zhang, Daimo Zhang, Xiaozhao Li, Juntao Feng

**Affiliations:** ^1^Department of Respiratory Medicine, National Key Clinical Specialty, Branch of National Clinical Research Center for Respiratory Disease, Xiangya Hospital, Central South University, Changsha, China; ^2^Department of Nephrology, Xiangya Hospital, Central South University, Changsha, China

**Keywords:** GLCCI1, glucocorticoid resistance, asthma, IRF1:GRIP1, IRF3:GRIP1

## Abstract

GLCCI1 plays a significant role in modulating glucocorticoid (GC) sensitivity in asthma. This project determines the underlying mechanism that GLCCI1 deficiency attenuates GC sensitivity in dexamethasone (Dex)-treated Ovalbumin (OVA)-induced asthma mice and epithelial cells through upregulating binding of IRF1:GRIP1 and IRF3:GRIP1. Dexamethasone treatment led to less reduced inflammation, airway hyperresponsiveness, and activation of the components responsible for GC activity, as determined by decreased GR and glucocorticoid receptor interacting protein 1 (GRIP1) expression but augmented IRF1 and IRF3 expression in GLCCI1^−/−^ asthmatic mice compared with wild type asthmatic mice. Moreover, the recruitment of GRIP1 to GR was downregulated, while the individual recruitment of GRIP1 to IRF1 and IRF3 was upregulated in GLCCI1^−/−^ Dex-treated asthmatic mice compared to wild type Dex-treated asthmatic mice. We also found that GLCCI1 knockdown reduced GR and GRIP1 expression but increased IRF1 and IRF3 expression in Beas2B and A549 cells. Additionally, GLCCI1 silencing increased the interactions between GRIP1 with IRF1 and GRIP1 with IRF3, but decreased the recruitment of GRIP1 to GR. These studies support a critical but previously unrecognized effect of GLCCI1 expression on epithelial cells in asthma GC responses by which GLCCI1 deficiency reduces the GR and GRIP1 interaction but competitively enhances the recruitment of GRIP1 to IRF1 and IRF3.

## Introduction

Approximately 10% of asthma patients have a poor response to glucocorticoid (GC) therapy (1–4). Even through distinct phenotype identification (5), no specific biomarkers are capable of predicting the clinical response to inhaled corticosteroid (ICS) in asthmatic patients have been discovered.

Tantisira et al. reported that a functional polymorphism in the GC-induced transcript 1 (GLCCI1) gene, rs37973, is closely related to the response to ICS in non-Hispanic white subjects with asthma (6). Moreover, other research has proposed that GLCCI1 is a pharmacogenetic determinant of the response to long-term ICS treatment in asthmatic patients that mainly accelerates pulmonary function decline in ICS treatment (7). Our own previous data showed that GLCCI1 variations are associated with asthma susceptibility and the ICS response in the adult Chinese Han population and that GLCCI1 expression might be affected by variations in GLCCI1 during the ICS response period (8). Additionally, GLCCI1 was positively localized on bronchial epithelial cells, and its variants could influence GC sensitivity (9). Therefore, GLCCI1 expressed on epithelial cells plays a crucial and important role in mediating the GC response in asthma, which might be beneficial for bio-identification, but the potential mechanism is not very clear.

During the GC response period, GR, a ligand-dependent transcription factor of the nuclear receptor superfamily, binds GC in the cytoplasm, dimerizes, and translocates to the nucleus, where it acts as a transcription factor (10). Glucocorticoid signaling via the GR, upon hormone binding, is recruited to genomic GC response elements (GREs) that regulate GC responses in therapeutic pathways (11). In terms of GC signaling, glucocorticoid receptor interacting protein 1 (GRIP1) has emerged as a unique family member, but unlike other p160s, it serves as a coactivator for all nuclear receptors, including GR, at “tethering” sites and facilitates GC-mediated repression of AP-1- and NF-κB-driven targets (12–15). Moreover, research has indicated that GRIP1, which is also a GR corepressor, can facilitate the anti-inflammatory effects of GCs *in vivo* by modulating the cytokines TNF α, IL-1β, and IFN γ (16). Notably, our own previous study reported that GLCCI1 deficiency in asthmatic mice led to GC resistance through reduced GR expression, but the detailed mechanism is not well known (17). Based on the role of the GR-GRIP1 pathway in GC responses and the observed decrease in GR expression induced by GLCCI1 deficiency in asthmatic mice under GC treatment, it is speculated that GLCCI1 regulates GC sensitivity through the GR-GRIP1 pathway.

Although originally identified as a nuclear receptor cofactor, GRIP1 was later shown to engage in physical and functional interactions with interferon regulatory factor (IRF) family members (IRF1 and IRF3) and function as an IRF3 coactivator in macrophages and an IRF1 coactivator in human airway smooth muscle cells (ASM) (18, 19). Recently, changes in IRF1 gene expression were found to act as a GC response marker in the airway and were even more predictive than NF-κB (20). Hence, we hypothesized that the interaction of IRF1 and IRF3 with GRIP1 participates in the mechanism by which GLCCI1 mediates the GR-GRIP1 pathway in the GC response.

The aim of this study was to explore the function of GLCCI1 expressed on epithelial cells in regulating GC responses via the GR-GRIP1 pathway in an Ovalbumin (OVA)-induced asthma model. Then, the latent function of IRF1 and IRF3 in competition with GR for GRIP1 binding under GLCCI1-deficient conditions was further investigated.

## Materials and Methods

### Animals and Development of an Asthma Model

All procedures performed on the animals were in compliance with the Chinese Council of Animal Care Guidelines, approved by the Central South University Animal Care Committee (No.201803478).

Female wild type (WT) and GLCCI1-knockout (GLCCI1^−/−^) C57BL/6 mice (6–8 weeks) were bred under SPF conditions in the Experimental Animal Center of Central South University, Changsha, Hunan, China. The homozygous GLCCI1^−/−^ mice were constructed as previously described (17).

Both WT and GLCCI1^−/−^ mice were randomly divided into the three following groups (*n* = 6): the control group (PBS), the mice in which were treated with PBS; the OVA-induced asthmatic group (OVA); and the Dex-treated OVA-induced asthmatic group (Dex+OVA). Ovalbumin-induced asthmatic mice and Dex-treated asthmatic mice were treated as previously described (17). Briefly, mice were sensitized with OVA (50 μg, Sigma, USA) plus aluminum hydroxide (2 mg, Sigma) in 0.2 ml of sterile saline on days 0, 7 and 14 by intraperitoneal (i.p.) injection. Moreover, all sensitized mice were exposed to aerosolized 5% OVA (wt/vol) for 30 mins every day from day 21 to 27. All mice in the control group received sterile PBS for sham sensitization and challenge. Mice in the Dex+OVA group were pre-treated with Dex (1 mg/kg) through i.p. injection every day 1 h before exposure to OVA aerosol. Furthermore, mice were sacrificed on day 28, and samples were collected for further detection.

### Cell Culture

Beas2B cells and A549 cells were obtained from Zhong Qiao Xin Zhou Biotechnology. Beas2B cells were cultured in complete bronchial epithelial cell medium (Zhong Qiao Xin Zhou Biotechnology, China), while A549 cells were cultured in RPMI 1640 with 10% FBS and 1% penstreptomycin in a 37°C incubator under 5% CO_2_.

Beas2B and A549 cells were separately seeded in six-well plates at a density of 3 × 10^5^ cells/well and then transfected for 24 h with 50 nM GLCCI1 siRNA (si-h-GLCCI1_001: GCTCTATGATCGTGATAAA) and with 50 nM negative control (NC) siRNA (Ribobio Co., Ltd., China) with ribo FECTTMCP transfection reagent (Ribobio Co., Ltd., Chia) according to the manufacturer's instructions.

### Measurement of Bronchial Responsiveness

Mouse airway responsiveness to methacholine was measured within 24 h after the last challenge using whole-body plethysmography (Buxco Electronics, Inc., USA). The mice were challenged for 2 min with aerosolized solutions of methacholine (0, 10, 20 and 30 mg/ml in normal saline), after which airway resistance (RL) was measured (21, 22). The results for each methacholine concentration are expressed as the percentage relative to baseline RL values after saline exposure.

### Lung Histopathology

The left lung was fixed in 4% paraformaldehyde for 48 h before being embedded in paraffin and then routinely processed. Serial 3 μm tissue sections were stained with hematoxylin and eosin (H&E) and periodic acid-Schiff (PAS) reagent. Morphological changes in the lung were observed under a light microscope.

### Immunoblotting

Mouse lung tissues were first ground with an electronic tissue grinder (Tiangen, China) and then incubated in RIPA lysis buffer (Beyotime, China) containing 1 mmol/L phenylmethanesulfonyl fluoride (PMSF) (MilliporeSigma) and protease inhibitor cocktail (Servicebio, China) for 30 min. The homogenates were centrifuged at 15,000 × g for 15 min at 4°C. The concentration of proteins in the supernatant fractions was determined with a BCA protein assay kit (Beyotime, China). Selected samples containing equal amounts of protein (50 μg) were subjected to 10% sodium dodecyl sulfate-polyacrylamide gel electrophoresis. Separated proteins were transferred to polyvinylidene fluoride (PVDF) membranes after electrophoresis. The membranes were blocked with 5% non-fat dry milk or 5% bull serum albumin (for phosphorylated proteins) dissolved in Tris-buffered saline with Tween-20 buffer for 1 h and then incubated at 4°C overnight with primary antibodies against GLCCI1 (1:500, Hangzhou Goodhere Biotechnology), GR (1:1,000, Cell Signaling Technology), GRIP1 (1:500, Santa Cruz), IRF1 (1:1000, Cell Signaling Technology), IRF3 (1:1000, Cell Signaling Technology), and MKP-1 (1:1000, Merck-Millipore), followed by incubation with horseradish peroxidase-conjugated secondary antibodies for 1 h at room temperature. The results are expressed as the ratio of the mean band density for the experimental groups to that for the control group after normalization to glyceraldehyde-3-phosphate dehydrogenase (GAPDH) as an internal control. The blots were visualized using an enhanced chemiluminescence detection system (Advansta, Menlo Park, USA).

### Coimmunoprecipitation

Coimmunoprecipitation (co-IP) using antibodies against IRF1, IRF3, GRIP-1, and GR was performed as described above. Proteins were collected from lung tissues, Beas2B cells and A549 cells with Cell lysis buffer for Western and IP (Beyotime, China) and a protease inhibitor cocktail (Thermo Fisher, USA). Samples from each group containing the same amount of protein were immunoprecipitated with anti-GRIP1 monoclonal antibody (mAb) overnight. Immunoprecipitates were transferred into PVDF membranes as described above and immunoblotted with anti-GR, anti-IRF1, and anti-IRF3 antibody, followed by incubation with HRP-conjugated anti-rabbit IgG Ab. All proteins were detected by using enhanced chemiluminescence detection.

### RNA Preparation and Real-Time Reverse Transcription-Polymerase Chain Reaction

Relative mRNA levels were detected using an ABI ViiA 7 real-time reverse transcription-polymerase chain reaction (RT-PCR) system (ABI, USA). Total RNA was extracted from the lung tissue of different mice, Beas2B and A549 cells with TRIzol reagent (Takara). After testing the concentration and integrity of RNA by detecting optical density, RNA was reverse transcribed to cDNA according to the manufacturer's instructions (Takara). Primers specific for GAPDH, GLCCI1, GR, GRIP1, IRF1, IRF3, CD38, GILZ, FKBP5, MKP-1, CCL2, CCL3, CCL4, and CCL7 used in RT-PCR were designed with Primer Premier 5.0 (Premier, Canada) and produced by Sangon Biotech (Shanghai, China), and their sequences are listed in Table 1. The RT-PCR mixtures contained cDNA, primers and SYBR mix in RNase-free double-distilled water. The RT-PCR conditions were as described in the instruction manual. The results are expressed as the ratio of the mean threshold cycle (Ct) value for experimental groups to that for the control group after normalization to GAPDH.

**Table 1:** Sequences of target genes for real-time PCR.

**Gene**	**Forward primer (5^**′**^-3^**′**^)**	**Reverse primer (5^**′**^-3^**′**^)**
GAPDH (mouse)	GGTTGTCTCCTGCGACTTCA	TGGTCCAGGGTTTCTTACTCC
GLCCI1 (mouse)	AGGCGAACCTCTTCTCTGGA	GTGAACATGAGGGTCCCGTG
GR (mouse)	GTGAGTTCTCCTCCGTCCAG	TACAGCTTCCACACGTCAGC
GRIP1 (mouse)	CTTGGCCTGACGGTATCGG	CCGCCTTGATGTAGTCGCC
CD38 (mouse)	TCTCTAGGAAAGCCCAGATCG	AGAAAAGTGCTTCGTGGTAGG
GILZ (mouse)	AACACCGAAATGTATCAGACCC	GTTTAACGGAAACCAAATCCCCT
FKBP5 (mouse)	GATGAGGGCACCAGTAACAATG	CAACATCCCTTTGTAGTGGACAT
IRF1 (mouse)	ATGCCAATCACTCGAATGCG	CCTGCTTTGTATCGGCCTGT
IRF3 (mouse)	GAGAGCCGAACGAGGTTCAG	CTTCCAGGTTGACACGTCCG
CCL2 (mouse)	AGGTCCCTGTCATGCTTCTGGG	CCTCATTGGGATCATCTTGCTGGTG
CCL3 (mouse)	CCTCGATGTGGCTACTTGGCAGC	CCTGAGAGTCTTGGAGGCAGCGA
CCL4 (mouse)	GTGCTCCAGGGTTCTCAGCACCAATG	GGTCAGGAATACCACAGCTGGCTTGG
CCL7 (mouse)	CCACATGCTGCTATGTCAAGA	ACACCGACTACTGGTGATCCT
GAPDH (human)	GGTGAAGGTCGGAGTCAACG	CAAAGTTGTCATGGATGACC
GLCCI1 (human)	CGGACCTCTAGTACAATAAGGCG	AGGTGTCTGAGTAGCTTTGTCT
GR (human)	ACAGCATCCCTTTCTCAACAG	AGATCCTTGGCACCTATTCCAAT
GRIP1 (human)	TGAGAGTCCCTACACTAAATCCG	ATTCCTCCCGATACCGTCAGA
CD38 (human)	CAACTCTGTCTTGGCGTCAGT	CCCATACACTTTGGCAGTCTACA
IRF1 (human)	CTGTGCGAGTGTACCGGATG	ATCCCCACATGACTTCCTCTT
IRF3 (human)	AGAGGCTCGTGATGGTCAAG	AGGTCCACAGTATTCTCCAGG
MKP1 (human)	ACCACCACCGTGTTCAACTTC	TGGGAGAGGTCGTAATGGGG

### Statistical Analysis

Data are shown as the mean ± SEM. The significant difference between two independent groups were evaluated by Student's *t*-test. Multigroup comparisons were carried out with one-way analysis of variance (ANOVA) followed by least significant difference (LSD)-test (GraphPad Prism software, GraphPad). The level of statistical significance was set at *p* < 0.05.

## Results

### Result 1 GLCCI1 Deficiency Leads to GC Insensitivity in OVA-Induced Asthmatic Mice

**Figure 1:**
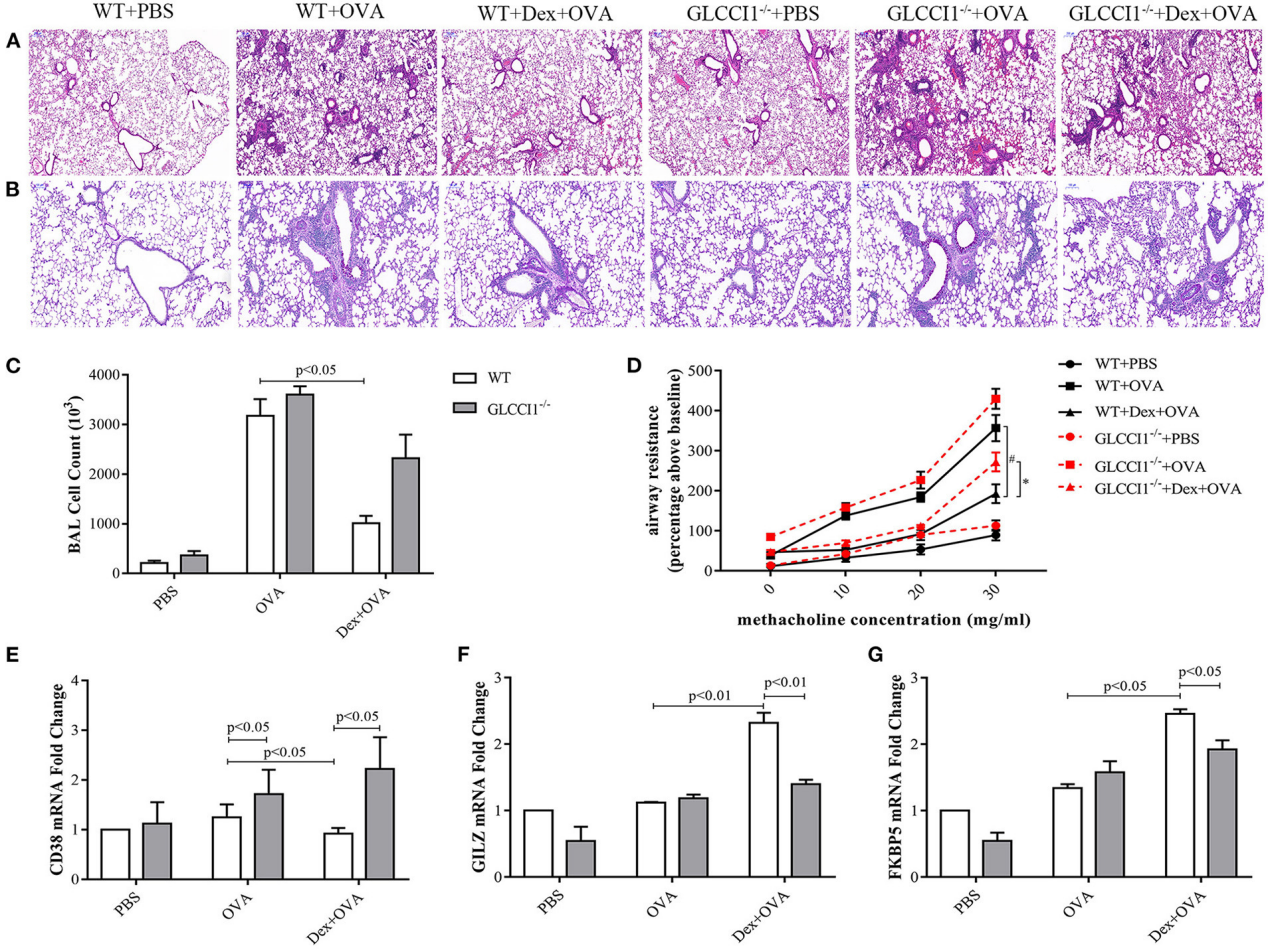
GLCCI1 deficiency leads to glucocorticoid insensitivity in OVA-induced asthmatic mice. **(A)** Representative H&E staining images (100 ×). **(B)** Representative PAS staining images (200 ×). **(C)** Whole BALF cell numbers in different groups. **(D)** Airway resistance detection. Result expressed as the airway resistance (percentage above baseline) indicating ratio with saline exposure. **(E–G)** RT-PCR analysis of CD38 **(E)**, GILZ **(F)**, and FKBP5 **(G)** mRNA expression in lung tissues. Results are presented as the mean ± SEM, *n* = 6 mice per group. *P < 0.05, ^#^*P* < 0.05. Intergroup differences as determined one-way ANOVA followed by LSD test.

We previously demonstrated the tendency for a decreased hydroprednisone response in GLCCI1^−/−^ mice with OVA-induced asthma when compared with WT mice with OVA-induced asthma (17). To further clarify and explore the potential function of GLCCI1 in the response to dexamethasone (Dex), asthmatic model mice were constructed in WT and GLCCI1^−/−^ mice treated with or without Dex. We first assessed lung inflammation in different mouse using H&E (Figure 1A), PAS staining (Figure 1B), and counting total cells in Bronchoalveolar lavage fluid (BALF) (Figure 1C). As shown in Figures 1A,B, H&E and PAS staining supported that obvious recruitment of inflammatory cells to the lungs, dense peribronchial infiltration, goblet hyperplasia was observed in WT and GLCCI1^−/−^ asthmatic mice. Moreover, total BALF cell numbers (Figure 1C) and airway resistance (Figure 1D) were also detected in WT and GLCCI1^−/−^ asthmatic mice. Dex treatment reduced lung damage, BALF total cells, airway resistance in WT asthmatic mice, however, Dex response in GLCCI1^−/−^ asthmatic mice is weak compared to WT asthmatic mice.

In addition, pro-inflammatory CD38 (Figure 1E) and GC-related genes such as GILZ and FKBP5 (Figures 1F,G) mRNA levels in lung tissues were detected by RT-PCR to further verify the role of GLCCI1 in the response to Dex. Interestingly, CD38 expression was remarkably increased in GLCCI1^−/−^ OVA-induced asthmatic mice and Dex-treated asthmatic mice compared with WT mice (Figure 1E). Furthermore, we observed increased expression of GILZ and FKBP5 in WT asthmatic mice treated with Dex compared to untreated WT asthmatic mice; however, there is no significant increase of GILZ and FKBP5 mRNA expression in GLCCI1^−/−^ asthmatic mice treated with Dex in comparison to untreated one (Figures 1F,G). All of the above evidence suggested that GLCCI1 deficiency led to Dex resistance in the OVA-induced asthma model (Figure 1).

### Result 2 GLCCI1 Silencing Results in GC Insensitivity in Human Epithelial Cells

**Figure 2:**
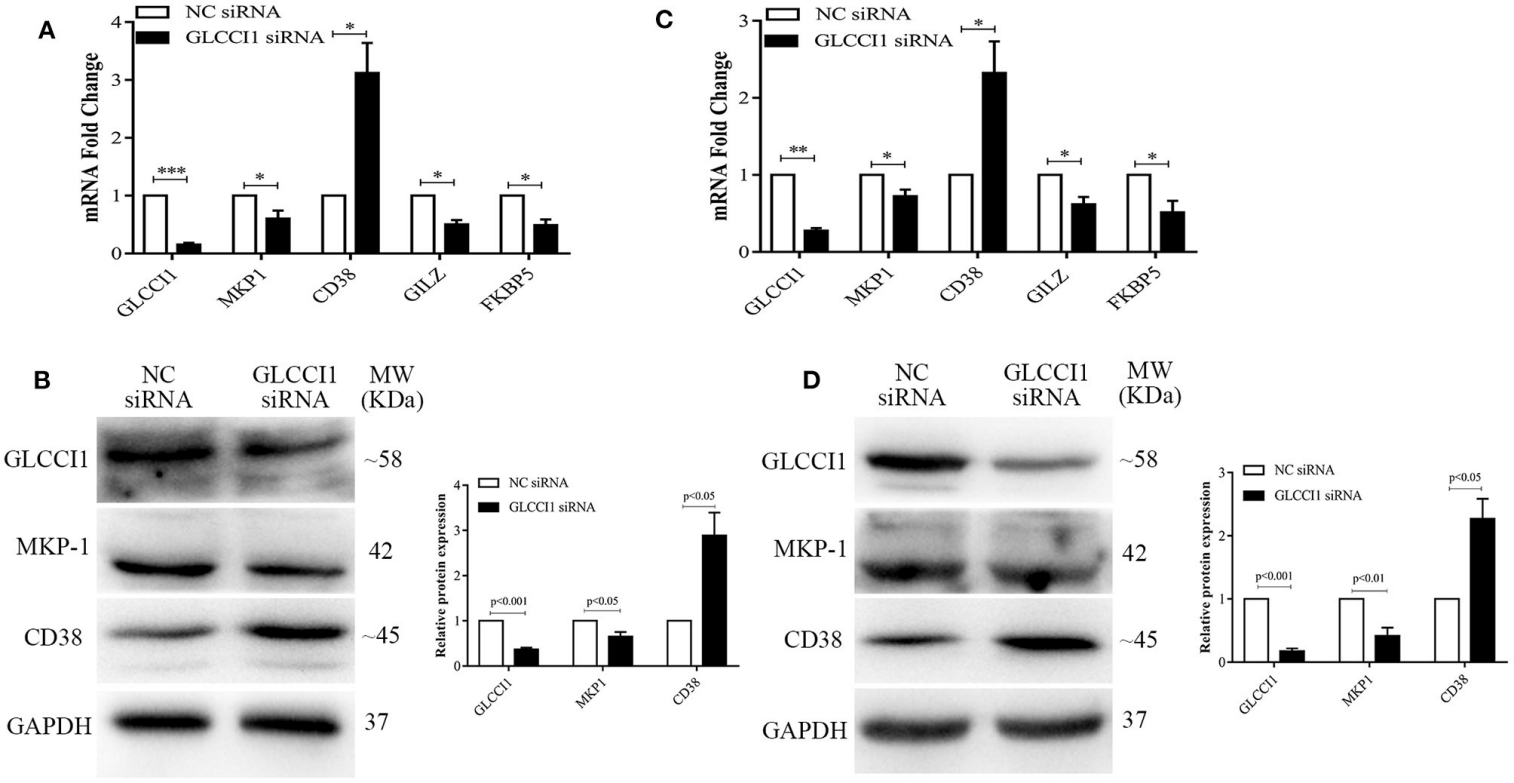
GLCCI1 deficiency results in glucocorticoid insensitivity in Beas2B and A549 cells. OVA and Dex were used to stimulate Beas2B and A549 cells in the presence or absence of GLCCI1 siRNA. **(A)** RT-PCR analysis of GLCCI1, MKP-1, and CD38, GILZ and FKBP5 mRNA expression in Beas2B cells. **(B)** Representative images and relative protein levels of GLCCI1, MKP-1, and CD38 assessed by WB in Beas2B cells. **(C)** RT-PCR analysis of GLCCI1, MKP-1, and CD38, GILZ and FKBP5 mRNA expression in A549 cells. **(D)** Representative images and relative protein levels of GLCCI1, MKP-1, and CD38 assessed by WB in A549 cells **(D)**. Data are presented as means ± SEM of more than three independent experiments. *P < 0.05, **P < 0.01, ***P < 0.001. Intergroup differences as determined by Student's *t*-test.

Chiba et al. reported that GLCCI1 was shown to be positively localized on bronchial epithelial cells and exhibit lower expression in asthma but higher expression with fluticasone stimulation (9). Therefore, Beas2B and A549 cells were used in this study to investigate the effect of GLCCI1 in epithelial cells. OVA and Dex were used to stimulate Beas2B (Figures 2A,B) and A549 cells (Figures 2C,D) in the presence or absence of GLCCI1 siRNA. Firstly, GLCCI1 siRNA showed good GLCCI1-silencing efficiency both in Beas2B (Figures 2A,B) and A549 cells (Figures 2C,D) assessed by RT-PCR and WB. Compared with that in the NC siRNA-treated group, increased CD38 mRNA and protein level, decreased MKP-1 at both mRNA and protein level and downregulated GILZ and FKBP5 expression was existed in GLCCI1 siRNA silenced Beas2B (Figures 2A,B) and A549 cells (Figures 2C,D). Taken together, GLCCI1 knocked down can lead to GC insensitivity in human epithelial cells.

### Results 3 GLCCI1 Deficiency Reduces GR-GRIP1 Binding Through Upregulating the Recruitment of GRIP1 to IRF1 and IRF3 in OVA-Induced Asthmatic Mice

**Figure 3:**
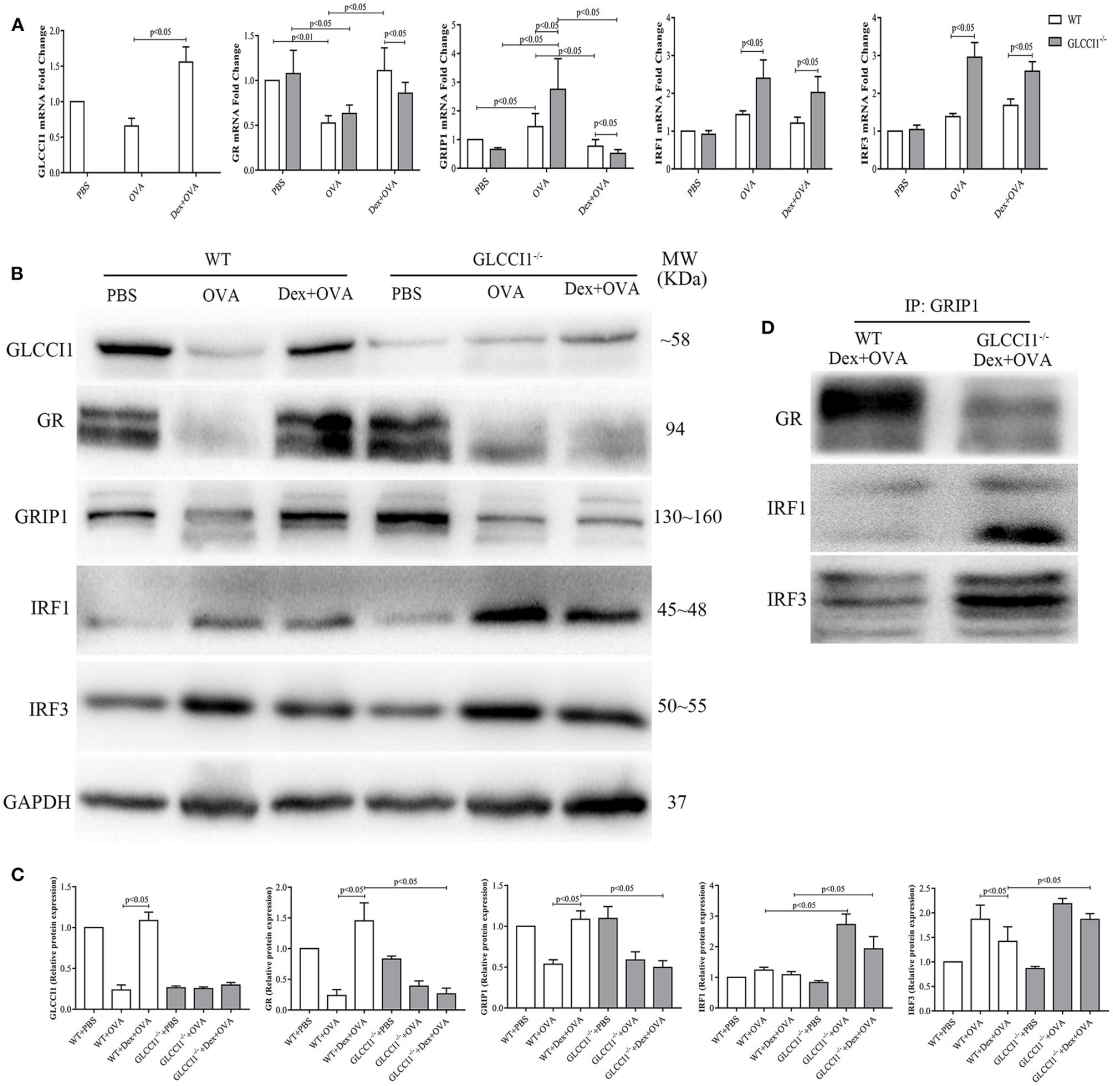
GLCCI1 deficiency suppresses GR-GRIP1 binding through increasing the recruitment of GRIP1 to IRF1 and IRF3 in OVA-induced asthmatic mice. **(A)** RT-PCR analysis of GLCCI1, GR, GRIP1, IRF1, and IRF3 mRNA expression in the lungs of mice from different groups. Representative images of protein expression detected by WB **(B)** and relative protein levels **(C)** of GLCCI1, GR, GRIP1, IRF1, and IRF3 in the lungs of mice in different groups. **(D)** Anti-GRIP1 mAb was used to capture GR, IRF1, and IRF3 in lung tissues from WT and GLCCI1^−/−^ Dex-treated mice to detect the individual binding of GRIP1 with GR, IRF1, and IRF3. Data are presented as the mean ± SEM, *n* = 6 mice per group. Independent groups were evaluated by Student's *t*-test, and intergroup differences as determined one-way ANOVA followed by LSD test.

We previously verified that hydroprednisone increased GLCCI1 expression, while GLCCI1 knockout reduced GR expression in hydroprednisone-treated asthmatic mice (17). To further explore the underlying mechanisms by which GLCCI1 influences the components responsible for GC activity, we detected GLCCI1, GR, and GRIP1 using RT-PCR (Figure 3A) and WB (Figures 3B,C). We observed higher GLCCI1, GR, and GRIP1 expression both at mRNA and protein level in Dex-treated WT asthmatic mice compared to untreated WT asthmatic mice. Most interestingly, as shown in Figures 3A–C, both GR and GRIP1 mRNA and protein expression was reduced in the lung tissues of GLCCI1^−/−^ Dex-treated asthmatic mice compared to WT Dex-treated asthmatic mice, which further supported our hypothesis that GLCCI1 can regulate the GR-GRIP1 pathway. Additionally, to observe the interaction between GR and GRIP1, anti-GRIP1 mAb was used to capture GR for assessment by co-IP with anti-GR. According to the results, the binding between GR and GRIP1 was downregulated in GLCCI1^−/−^ Dex-treated asthmatic mice compared to WT Dex-treated asthmatic mice (Figure 3D).

To further explore whether GLCCI1 reduces the interaction of GRIP1 with GR through the IRF family, we first assessed IRF1 and IRF3 expression in lung tissues from WT and GLCCI1^−/−^ asthmatic and Dex-treated asthmatic mice. IRF1 and IRF3 mRNA and protein expression was increased in GLCCI1^−/−^ Dex-treated asthmatic mice compared to WT Dex-treated asthmatic mice (Figures 3A–C). Furthermore, co-IP was used to examine GRIP1 recruitment to IRF1 and IRF3. As displayed in Figure 3D, the interactions between IRF1 with GRIP1 and IRF3 with GRIP1 were increased in Dex-treated GLCCI1^−/−^ asthmatic mice compared with WT Dex-treated asthmatic mice.

### Result 4 GLCCI1 Silencing in Epithelial Cells Enhances the Binding of IRF1 and IRF3 With GRIP1 While Reducing GR and GRIP1 Binding

**Figure 4:**
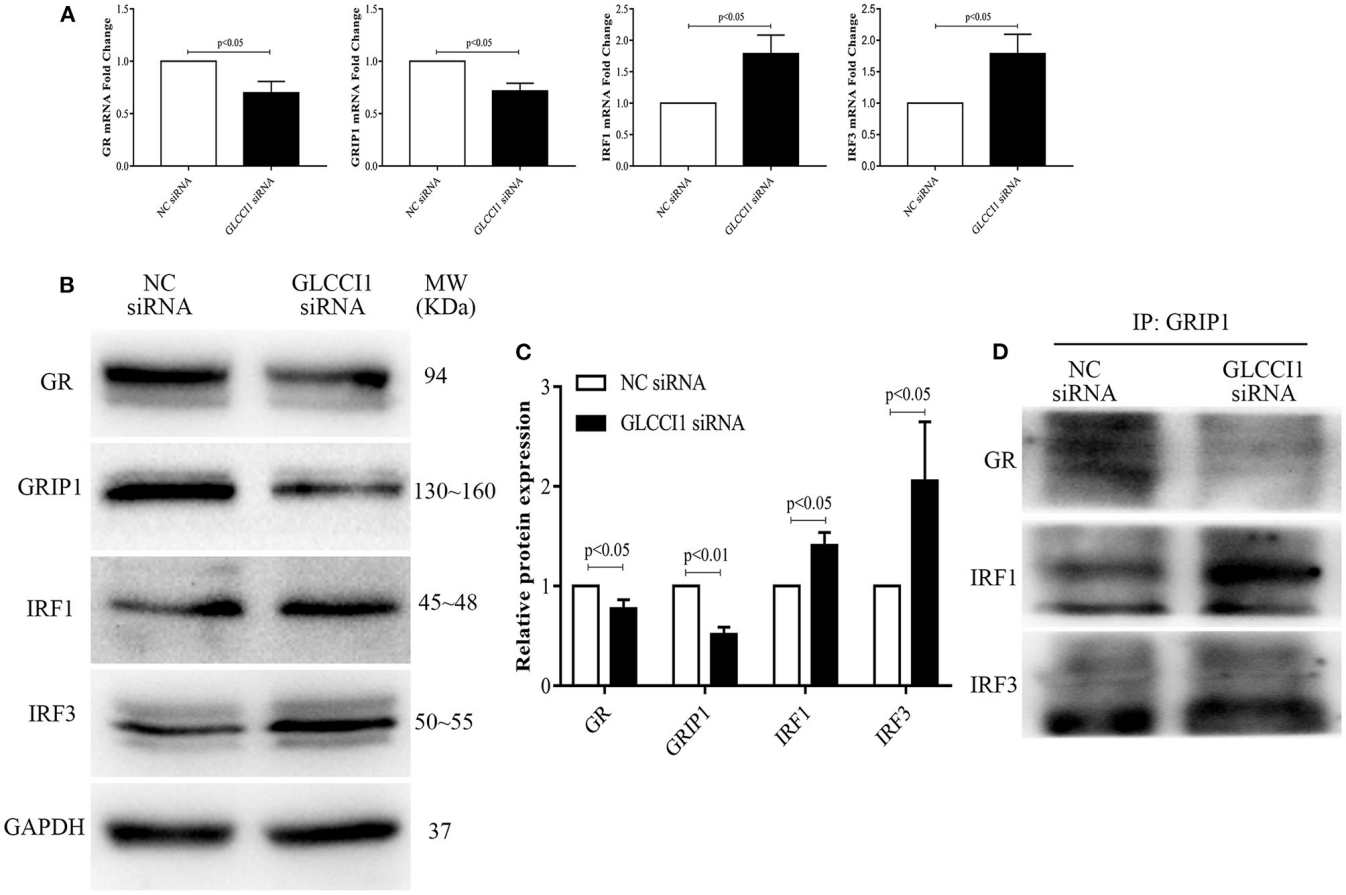
GLCCI1 enhances the binding of IRF1 and IRF3 with GRIP1 and reduces GR and GRIP1 binding in Beas2B cells. Beas2B cells were treated with OVA and Dex in the presence and absence of GLCCI1 siRNA. **(A)** RT-PCR anylysis of GR, GRIP1, IRF1, and IRF3 mRNA expression. Representative images of protein expression **(B)** and relative protein levels **(C)** of GR, GRIP1, IRF1, and IRF3. **(D)** Anti-GRIP1 antibody was used to capture GR, IRF1, and IRF3 to detect the individual binding of GRIP1 with GR, IRF1, and IRF3. Data are presented as means ± SEM of more than three independent experiments. Intergroup differences as determined by Student's *t*-test.

**Figure 5:**
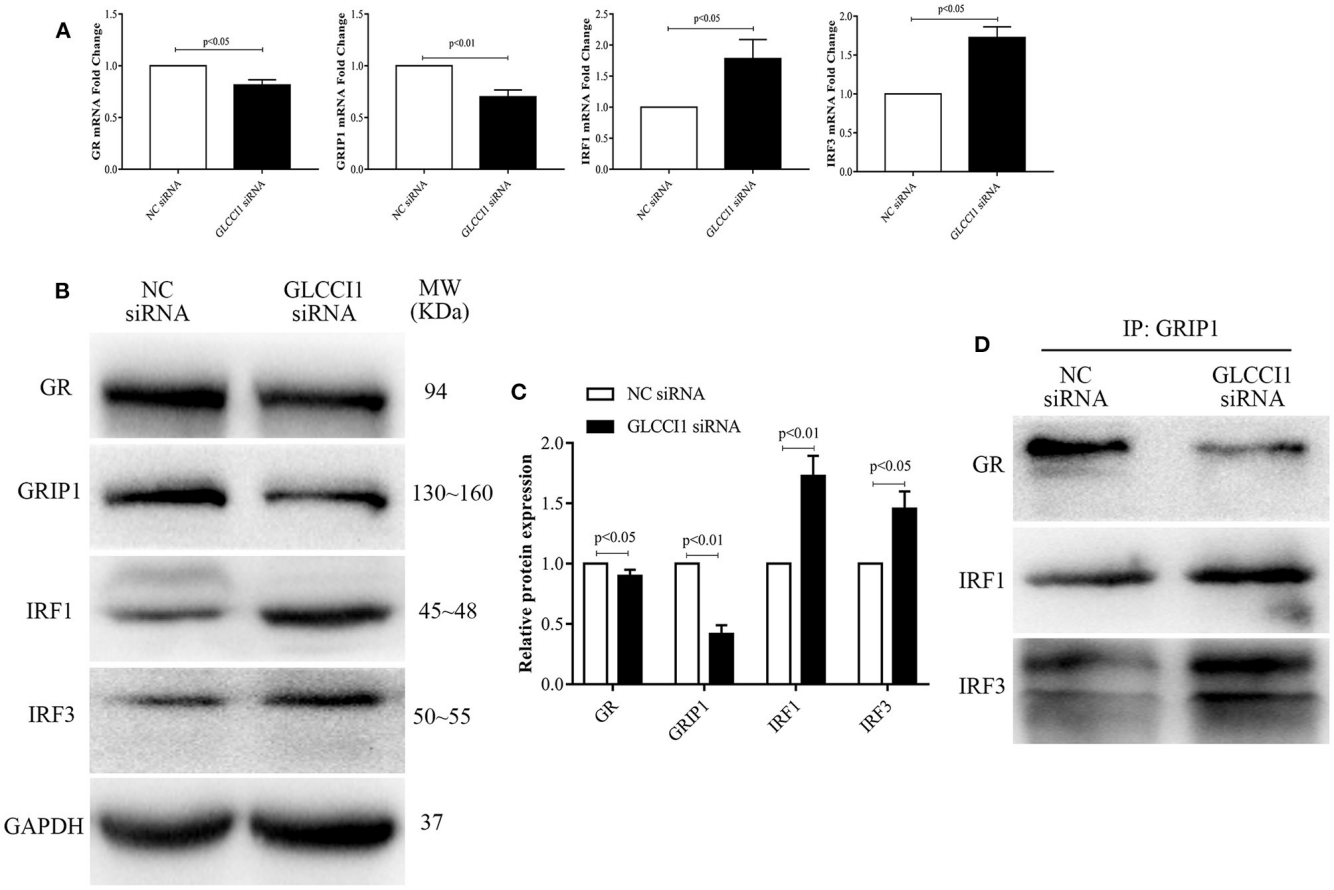
GLCCI1 heightens the binding of IRF1 and IRF3 with GRIP1 and reduces GR and GRIP1 binding in A549 cells. A549 cells were treated with OVA and Dex in the presence and absence of GLCCI1 siRNA. **(A)** RT-PCR analysis of GR, GRIP1, IRF1, and IRF3 mRNA expression. Representative images of protein expression **(B)** and relative protein levels **(C)** of GR, GRIP1, IRF1, and IRF3. **(D)** Anti-GRIP1 antibody was used to capture GR, IRF1, and IRF3 to detect the individual binding of GRIP1 with GR, IRF1, and IRF3. Data are presented as means ± SEM of more than three independent experiments. Intergroup differences as determined by Student's *t*-test.

We also analyzed the role of GLCCI1 in OVA- and Dex-treated Beas2B (Figure 4) and A549 (Figure 5) cells. Beas2B cells and A549 cells that had been transfected with NC siRNA or GLCCI1 siRNA were treated with OVA and/or Dex. Similar to the results of *in vivo* experiments, GLCCI1 siRNA reduced GR and GRIP1 expression while increasing IRF1 and IRF3 expression at both mRNA and protein level in Beas2B cells (Figures 4A–C) and A549 cells (Figures 5A–C) under OVA and Dex stimulation compared to that in NC siRNA-transfected cells under OVA and Dex stimulation.

Additionally, the recruitment of GRIP1 to IRF1 and IRF3 was augmented, while the binding of GRIP1 and GR was reduced in Beas2B (Figure 4D) and A549 cells (Figure 5D) in the presence of GLCCI1 siRNA under OVA and Dex stimulation. Together with data on the role of GLCCI1 in the response to Dex from above *in vivo* and *in vitro* experiments, these results indicated that GLCCI1 deficiency can upregulate IRF1 and IRF3 thus favoring their competitive interaction with GRIP1, inhibiting the effect of GRIP1 recruitment to GR in Dex-treated asthmatic mice and Dex-treated bronchial epithelial cells, which leads to GC resistance.

### Result 5 GLCCI1 Deficiency Disturbs Chemokine Dysfunction in OVA-Induced Asthmatic Mice

**Figure 6:**
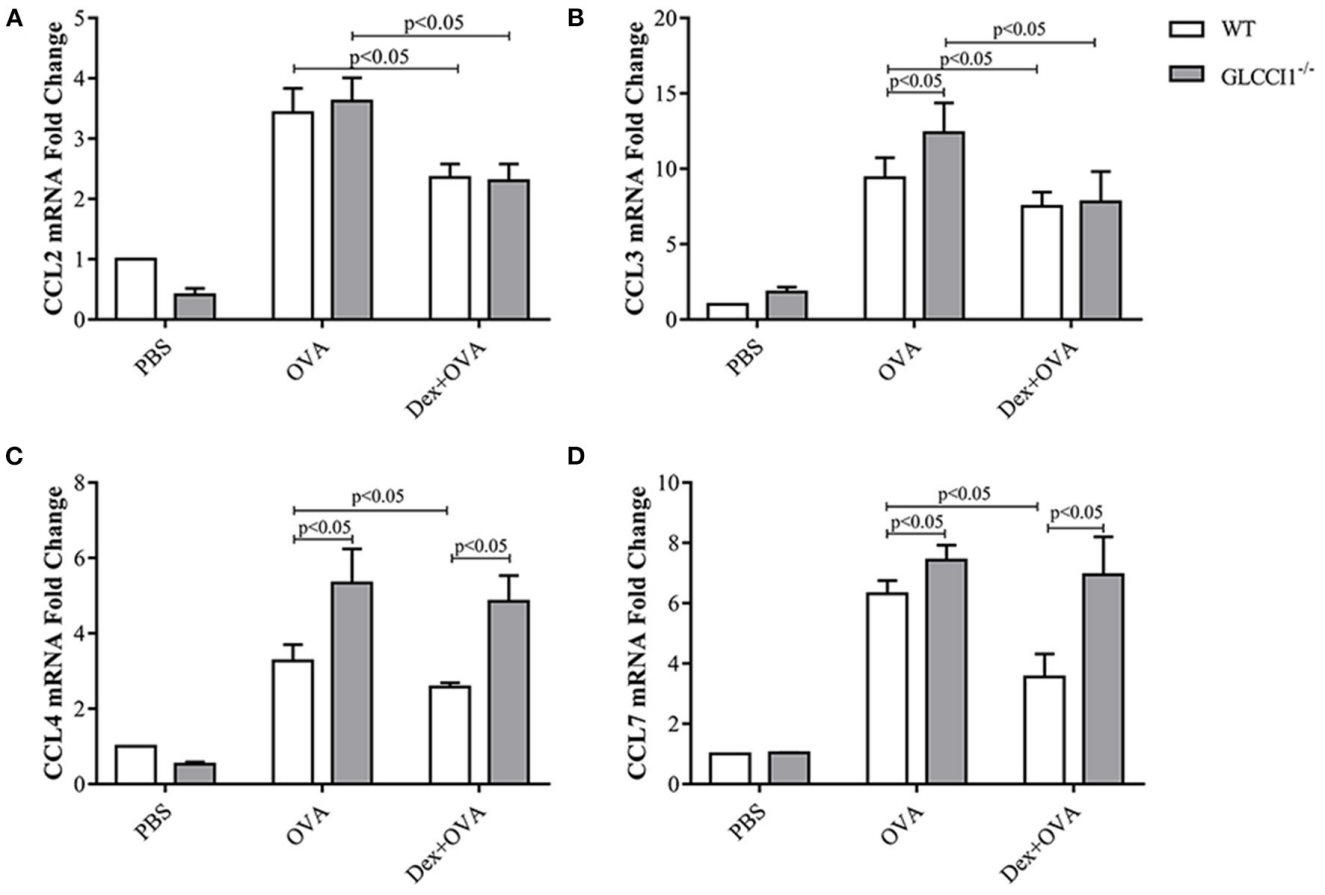
GLCCI1 mediates chemokine dysfunction in OVA-induced asthmatic mice. RT-PCR analysis of CCL2 **(A)**, CCL3 **(B)**, CCL4 **(C)**, CCL7 **(D)** mRNA levels in lung tissues from mice in different groups. Data are presented as the mean ± SEM, *n* = 6 mice per group. Intergroup differences as determined one-way ANOVA followed by LSD test.

To ascertain the mechanisms underlying GLCCI1-mediated regulation of the effects of GCs on chemokines, we began by assessing asthma-related pro-inflammatory chemokines (CCL2, CCL3, CCL4, and CCL7) in lung tissues by RT-PCR (Figures 6A–D). As shown in Figure 6, OVA induced CCL2, CCL3, CCL4, and CCL7 expression in both WT and GLCCI1^−/−^ asthmatic mice, but CCL3, CCL4, and CCL7 expression was even higher in GLCCI1^−/−^ asthmatic mice than in WT asthmatic mice, which indicated that GLCCI1 deficiency led to severe OVA-induced lung inflammation through regulating chemokine dysfunction. Additionally, Dex treatment of WT asthmatic mice reduced the expression of the above chemokines. Notably, Dex treatment of GLCCI1^−/−^ asthmatic mice also downregulated CCL2 and CCL3 expression but had no influence on CCL4 and CCL7 expression, which suggested that CCL4 and CCL7 may have a potential role in GLCCI1 deficiency-induced GC insensitivity.

## Discussion

**Figure 7:**
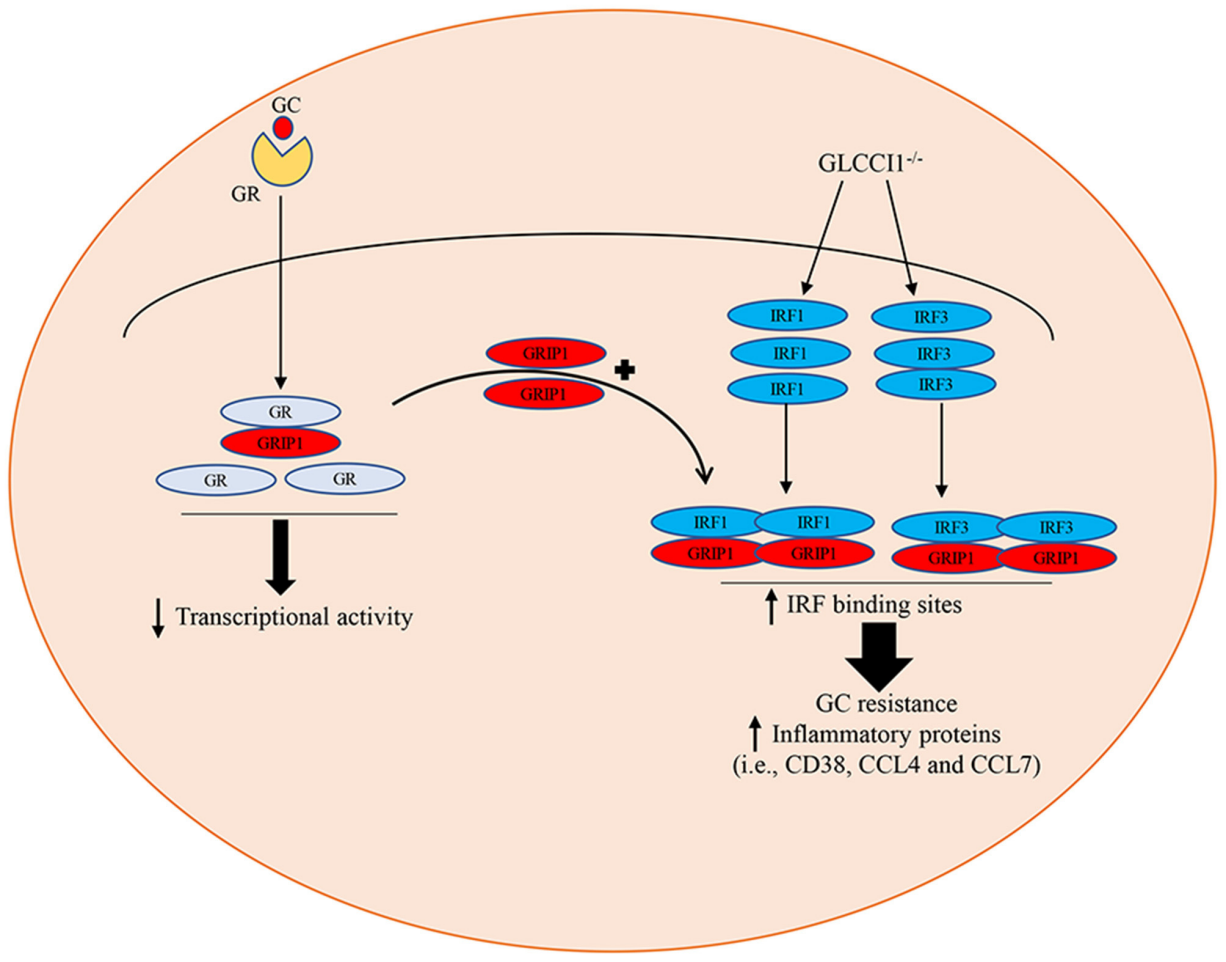
Schematic summary of the role of GLCCI1 in GC-treated OVA-induced asthma. GLCCI1 expression on epithelial cells in GC responses in asthma by which GLCCI1 deficiency reduces the GR and GRIP1 interaction but competitively enhances the recruitment of GRIP1 to IRF1 and IRF3.

Our previous research indicated that GLCCI1 deficiency can result in GC insensitivity in asthmatic patients and mice, but the potential mechanism is not very clear (8, 17, 23). The present project confirms that the loss of GLCCI1 expression leads to GC insensitivity through downregulating the GR-GRIP1 pathway, which is associated with the increased interaction between IRF1 and GRIP1 and between IRF3 and GRIP1 in asthma (Figure 7). These results support previous findings that GLCCI1 deficiency leads to GC resistance and further reveal a more detailed mechanism, which might be of potential value to explain, at least in part, the loss of corticosteroid efficacy in uncontrolled severe asthma.

Consistent with research by Tantisira (6), our own previous studies identified GLCCI1 as a novel pharmacogenetic determinant of the response of asthmatic patients and asthmatic mice to ICS (8, 17). As expected, both WT and GLCCI1^−/−^ asthmatic mouse models exhibited lung damage and inflammatory cell infiltration, airway resistance. Notably, Dex-treated WT asthmatic mice exhibited distinctly reduced airway resistance and decreased airway and lung inflammation, while the effect of Dex in GLCCI1^−/−^ asthmatic mice was lacking, with a smaller reduction in airway resistance and lung damage observed. Additionally, decreased CD38 expression in WT Dex-treated asthmatic mice compared to GLCCI1^−/−^ Dex-treated asthmatic mice indicated that the Dex response was reduced in GLCCI1-deficient mice. Additionally, an obvious increase in the expression of GILZ and FKBP5, which are closely related to the GC response in WT Dex-treated asthmatic mice, was not observed in GLCCI1^−/−^ Dex-treated asthmatic mice. Taken together, these results indicated that the anti-inflammatory effects of Dex were less efficacious in GLCCI1^−/−^ asthmatic mice. Consistent with the above phenomenon, our previous study reported that GLCCI1 deficiency could suppress GR and MKP-1 activation, with increased phosphorylation of p38 MAPK and GC insensitivity (17). However, whether GLCCI1 mediates GC responses through regulating GC-GR complex formation and, if so, the exact mechanism remains unclear.

GR, which belongs to the steroid hormone receptor subfamily of the nuclear receptor superfamily, can regulate GC function, and the most specific mechanism of this function dictates that GR might elicit transcriptional changes by recruiting multiple cofactors from the p160 family. Moreover, GRIP1, a cofactor of the p160 family, was originally described as a coactivator and can also act as a GR ligand-dependent corepressor of GR-NF-κB complexes (13, 16, 24). In addition, Rebecca et al. further reported that GR-GRIP1 uses distinct mechanisms to repress inflammatory genes at different stages of the transcription cycle (25). Taken together, these data present that it is worth investigating whether the GR-GRIP1 complex plays a vital role in GLCCI1-associated GC function.

To further investigate whether GLCCI1 deficiency regulates the GR-GRIP1 complex, we detected GR, GRIP1 and GR-GRIP1 binding in lung tissues of WT and GLCCI1^−/−^ Dex-treated asthmatic mice. As shown by the results, GLCCI1 deficiency led to the decreased expression of GR and GRIP1 in lung tissues, as detected by RT-PCR and WB. Additionally, our data illustrated decreased binding of GRIP1 to GR in GLCCI1^−/−^ Dex-treated asthmatic mice than in WT Dex-treated asthmatic mice. In short, global GLCCI1 knockout in Dex-treated asthmatic mice could reduce GR and GRIP1 expression, decreasing the less binding of GRIP1 to GR, which finally reduced Dex anti-inflammatory responses and led to GC insensitivity in asthma.

GRIP1 has previously been shown to interact with IRF3 and act as a corepressor for GR in macrophages, and increased competition between GR and IRF3 for GRIP1 antagonizes the IRF3-mediated transcription of inflammatory genes (14, 18). Additionally, Reena et al. reported that abnormally increased IRF-1 levels led to not only increased IRF1-dependent inflammatory proteins but also increased competition of GR for GRIP1 in ASM cells and decreased GC function (19). Combined with William's research on the evaluation of Gene Expression Omnibus data sets from ASM cells, IRF1 was verified to promote GC resistance in airway structure cells in both normal human bronchial epithelial cells and peripheral blood mononuclear cells (PBMCs) (20). In summary, it is speculated that the IRF family (IRF1 and IRF3) and GR and GRIP1 have opposite functions in the GC mechanism.

To further clarify our hypothesis, IRF1 and IRF3 expression in lung tissues was examined. As the results showed, IRF1 and IRF3 expression, assessed by RT-PCR and WB, was reduced in WT and GLCCI1^−/−^ Dex-treated asthmatic mice; however, IRF1 and IRF3 levels were higher in GLCCI1^−/−^ Dex-treated asthmatic mice than in WT Dex-treated asthmatic mice. Increased IRF1 and IRF3 expression might take part in GLCCI1 deficiency-induced GC insensitivity in asthma. To further verify the above hypothesis, anti-GRIP1 mAb was used to individually capture IRF1 and IRF3 in lung tissues from the Dex-treated group for assessment by co-IP. Our results indicated that binding between the IRF1-GRIP1 complex and IRF3-GRIP1 complex was increased in GLCCI1^−/−^ Dex-treated asthmatic mice compared with WT Dex-treated asthmatic mice. The co-IP data further support our hypothesis that GLCCI1 deficiency leads to GC insensitivity by increasing IRF1-GRIP1 complex and IRF3-GRIP1 complex binding while decreasing GR-GRIP1 binding.

Recent research reported that GLCCI1 is highly expressed in the airways, especially in epithelial cells, after ICS treatment and that GLCCI1 is expressed in cultured epithelial cells harboring GLCCI1 variants, resulting in worse pulmonary function (9). Additionally, the ability of Dex to modulate GR transcription in airway epithelial cells was found to coincide with its potency to resolve allergic airway inflammation (26). According to new insight into the significance of epithelial cells in GC sensitivity in asthma and the high level of GLCCI1 expression in epithelial cells, we chose to continue exploring the detailed mechanism of GLCCI1 in GC function in epithelial cells. Therefore, to determine whether GLCCI1 deficiency mediates GC function in asthmatic conditions through epithelial cells, Beas2B and A549 cells with or without GLCCI1 siRNA stimulation were used. In this study, GLCCI1 siRNA stimulation decreased GC-induced MKP-1, GILZ, and FKBP5 gene expression and increased CD38 gene expression in Beas2B and A549 cells under OVA and Dex treatment, which suggested that GLCCI1 indeed regulates GC function in epithelial cells. Moreover, GR and GRIP1 expression was downregulated, while IRF1 and IRF3 expression was increased in Beas2B and A549 cells stimulated with GLCCI1 siRNA compared to those stimulated with NC siRNA. Moreover, GLCCI1 downregulation also decreased GR-GRIP1 complex binding but increased IRF1-GRIP1 and IRF3-GRIP1 complex binding. All of the above data at the cellular level support the notion that GLCCI1 deficiency can mediate GC function in epithelial cells through decreasing the GR-GRIP1 pathway and increasing the recruitment of IRF1 and IRF3 by GRIP1.

Additionally, we observed obviously increased CCL3 and CCL4 expression in GLCCI1^−/−^ asthmatic mice compared to WT asthmatic mice, which may indicate that GLCCI1 deficiency can lead to severe lung inflammation. More interestingly, CCL2, CCL3, CCL4, and CCL7 were reduced in WT asthmatic mice treated with Dex, but CCL4 and CCL7 expression did not seem to change in GLCCI1^−/−^ Dex-treated asthmatic mice. Therefore, GC resistance led by GLCCI1 deficiency may be closely associated with CCL4 and CCL7. Combined, these results suggest that GLCCI1 deficiency-induced chemokine production can be attributed to the GRIP1 pathway, and this finding represents a future direction.

In summary, we have carried out an extremely potent investigation of the GLCCI1 gene, which, to the best of our knowledge, is a GC-regulated gene. Analysis of the mechanisms to explain the loss of GCs efficacy indicated that GCs partially regulate their anti-inflammatory effects through GLCCI1/GR-GRIP1 signaling. This study reveals a potential mechanism for mutual antagonism between IRF-1 and GR and IRF3 and GR in their interaction with GRIP1 with or without GLCCI1 expression in asthmatic mice treated with Dex, Beas2B cells and A549 cells. GLCCI1 deficiency in Dex-treated asthmatic mice, Beas2B cells and A549 cells might have limited the interaction of GRIP1 with GR but increased IRF1-GRIP1 and IRF3-GRIP1 complex binding, which dramatically reduced GC function. Our study also shows that GLCCI1 deficiency not only regulated the GC-GR pathway and IRF-GRIP1 pathway but also upregulated the chemokines CCL4 and CCL7 with decreased steroid responsiveness.

## Data Availability Statement

The raw data supporting the conclusions of this article will be made available by the authors, without undue reservation.

## Ethics Statement

The animal study was reviewed and approved by Central South University Animal Care Committee.

## Author Contributions

JF and XL conceived and designed the study and finalized the manuscript. XH conducted the experiments, analyzed data, and edited the manuscript. SD, LL, and FY conducted part of *in vitro* experiments. YJ, HG, YZ, and DZ performed *in vivo* experiments and analyzed data. All authors read and approved the final version of the manuscript.

## Conflict of Interest

The authors declare that the research was conducted in the absence of any commercial or financial relationships that could be construed as a potential conflict of interest.

## Publisher's Note

All claims expressed in this article are solely those of the authors and do not necessarily represent those of their affiliated organizations, or those of the publisher, the editors and the reviewers. Any product that may be evaluated in this article, or claim that may be made by its manufacturer, is not guaranteed or endorsed by the publisher.
